# Therapeutic Decision Making in Hepatocellular Carcinoma According to Age and Child–Pugh Class: A Nationwide Cohort Analysis in South Korea

**DOI:** 10.1155/2021/6640121

**Published:** 2021-01-04

**Authors:** Sunmin Park, Chai Hong Rim, Young Kul Jung, Won Sup Yoon

**Affiliations:** ^1^Department of Radiation Oncology, Korea University Ansan Hospital, Ansan, Gyeonggi-do, Republic of Korea; ^2^Division of Gastroenterology and Hepatology, Department of Internal Medicine, Korea University Ansan Hospital, Ansan, Gyeonggi-do, Republic of Korea

## Abstract

**Background:**

We sought to analyze the preferred treatment modality by age and liver function in South Korea.

**Methods:**

The Korean Liver Cancer Study Group randomly extracted the data of patients with hepatocellular carcinoma (HCC) enrolled in the Korean Central Cancer Registry from 2008 to 2014 from approximately 50 hospitals nationwide. After excluding distant and lymphatic metastases, the treatment preference for patients with a single lesion (excluding PVT (portal vein thrombosis), hepatic vessels, and bile duct invasion) and with PVT was evaluated in 7559 patients. Patients were grouped by age, and baseline liver function was divided based on the Child–Pugh class (CPC) *A*, *B*, and *C*.

**Results:**

For a single HCC, the majority of patients selected transarterial therapy as the initial treatment, followed by surgical resection and local ablative therapy. The surgical resection rate decreased significantly with age (*p* < 0.001), and the transarterial therapy rate significantly increased (*p* < 0.001). For CPC C, liver transplantation was significantly increased to 11.5%, and 36.3% of patients received no treatment. In HCC with PVT, the transarterial therapy rate was the highest, followed by the rate of abandonment of treatment. The proportion of no treatment significantly increased with age (*p* < 0.001). In CPC C, transarterial therapy and systemic therapy were attempted in 15.4% and 5.8% of patients, respectively.

**Conclusions:**

Age and liver function have a significant impact on the therapeutic decision-making of HCC patients in Korea. In unfavorable conditions, surgical resection was less favored in patients with single tumors, and no treatment was preferred in patients with PVT.

## 1. Introduction

Various guidelines for hepatocellular carcinoma (HCC) currently recommend standard and alternative treatment methods according to the tumor stage using characteristics such as tumor size, number of tumors, and degree of invasion of the major structures [1–6]. Patients with very early- to early-stage HCC should be considered for potentially curative options such as surgical resection, radiofrequency ablation (RFA), and transplantation. In patients with portal vein invasion belonging to the advanced stage (Barcelona clinic liver cancer (BCLC) C), systemic treatments including sorafenib are recommended as the standard treatment. Although the treatment of HCC is suggested by several guidelines as described above, various situations make HCC difficult to treat.

Together with the aging of the population in South Korea, the number of older people with cancer has increased considerably in the last decade [7]. The most common cause of HCC in East Asian countries, including China and Korea, is chronic hepatitis B virus (HBV) infection; patients in these countries tend to be diagnosed with more advanced disease [8, 9], which eventually leads to a poor Child–Pugh class (CPC). According to recent statistics in South Korea, more than 1 in 4 HCC patients are vulnerable; in that, 24.8% of cases occurred in people in their 70s [10] and 28.4% developed the CPC *B* or *C* [11]. However, many clinical trials exclude these groups of geriatrics, as well as those with unfavorable liver function, and evidence to guide treatment of these patients remains limited. In this situation, strategies for vulnerable HCC patients can be suggested by not only the guidelines but also the previous experience of actual practice.

Korean trends in treatment have not been previously published. Based on the cohort of the HCC registry project, this study was designed to investigate the preferred treatment methods in actual clinical practice. We specifically focused on the age and basal liver status of patients.

## 2. Materials and Methods

### 2.1. Database Source

Since 1980, the Korean Ministry of Health and Welfare has funded the Korean Central Cancer Registry (KCCR). The Korean Liver Cancer Study Group (KLCSG) and the National Cancer Center extracted HCC patient records from the KCCR to settle the mother population, which have been assigned codes of C22.0 according to the ICD-10. The studies performed by the KLCSG between 2008 and 2014 in South Korea were randomly selected among all hospitals nationwide that registered HCC patients in the KCCR. At least, one hospital was selected from all 16 administrative districts in South Korea, and the probability proportional extraction method, which is more likely to select hospitals with a large number of registered patients, was used. Through the three projects, the records of 15,078 patients who were initially diagnosed with HCC between 2008 and 2014 were sampled. Data on dates and causes of mortality were obtained from the Korean Statistical Information Service. The date of the first diagnosis of HCC was provided in the KCCR data.

### 2.2. Study Cohort

Of the total 15078 patients, we excluded patients under 18 years of age with initial lymph node metastases and/or distant metastases. For the analyses of single lesions, portal vein thrombosis (PVT), hepatic vessels, and bile duct invasive patients were additionally excluded. A total of 7559 patients were enrolled and analyzed, and subcohorts of single lesion and PVT were constructed. Age groups were classified as <59, 60–69, and ≥70 years. Baseline liver function was divided into CPC A, B, or C. The groups of single lesions were divided into ≤2 cm and >2 cm by the size of the tumor, and patients with PVT were analyzed in total. The preference of treatment was analyzed by the age group and baseline liver function status. Treatments were classified into surgical resection, liver transplantation, local ablative therapy (including mainly RFA, alcohol injection, and other local ablation), transarterial therapy (including mainly TACE with gelfoam, beads and lipiodol without gelfoam, transarterial chemoinfusion, and radioembolization), systemic chemotherapy (sorafenib and other systematic therapy), radiation therapy (RT), and no treatment according to the initial presentation of treatment.

### 2.3. Outcome Assessment and Statistical Analysis

The changes in treatment patterns were assessed. A Chi-square test was conducted to analyze the difference in treatment preference in terms of age and liver function. All statistical tests were performed using SPSS (version 22; IBM, Armonk, NY).

### 2.4. Ethical Consideration

The data source of the present study is public open data without personal identification information from the KCCR. Institutional review board approval was waived; in all other respects, we recognized and adhered to the World Medical Association Declaration of Helsinki.

## 3. Results

### 3.1. Patient Characteristics

The median age of the patients was 60.0 years (range, 21–98), and 5838 patients (77.2%) were men in a total of 7559 patients. CPC of *A*, *B*, and *C* were seen in 5855 (77.5%), 1422 (18.8%), and 282 (3.7%) patients, respectively (Table 1). Among them, our single lesion and PVT-positive cohorts were satisfied by 4633 patients and 625 patients, respectively. CPC B patients accounted for 667 (14.4%) patients in single lesion and 235 (38.6%) patients in PVT-positive cohorts. In all patient cohorts with CPC *B*, we analyzed treatment patterns in 1394 patients excluding 28 with missing data, and 55.2% of patients selected transarterial therapy as the initial treatment, followed by no treatment (24.7%) and systemic chemotherapy (10.6%). In a single lesion cohort with CPC B, transarterial therapy accounted for 54.4%, followed by local ablative therapy (17.7%) and no treatment (16.0%). Of the PVT-positive patients with CPC B, the proportion of patients with ‘no treatment' was 46.8%, followed by transarterial therapy (34.5%) and systemic chemotherapy (14.5%).

### 3.2. Single Lesion

**Figure 1:**
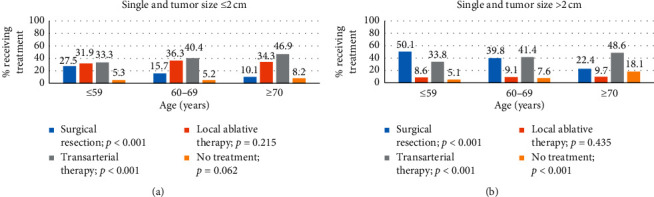
Proportion of the treatments performed for a single lesion according to age group. (a) Tumor size ≤2 cm for surgical resection (*p* < 0.001), local ablative therapy (*p*=0.215), transarterial therapy (*p* < 0.001), and no treatment (*p*=0.062). (b) Tumor size >2 cm for surgical resection (*p* < 0.001), local ablative therapy (*p*=0.435), transarterial therapy (*p* < 0.001), and no treatment (*p* < 0.001).

**Figure 2:**
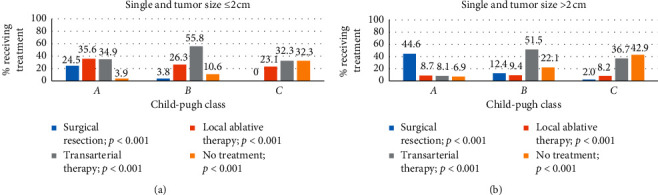
Proportion of the treatments performed for a single lesion according to Child–Pugh class. (a) Tumor size ≤2 cm for surgical resection, local ablative therapy, transarterial therapy, and no treatment (all *p* < 0.001). (b) Tumor size >2 cm for surgical resection, local ablative therapy, transarterial therapy, and no treatment (all *p* < 0.001).

Of the total 4633 single lesions, 43.6% were ≤2 cm and 56.4% were >2 cm. In the single lesion group, 39.1% of patients selected transarterial therapy as the initial treatment, followed by surgical resection (31.4%) and local ablative therapy (19.8%). Compared to the standard management (surgical resection or local ablation therapy) in most guidelines, transarterial therapy is preferred in patients ≥70 years of age and with CPC *B* and *C* (Table 2), and RT had the lowest rate in single-lesion patients (0.5%). In terms of age, the proportion of surgical resection significantly decreased (39.6% vs. 28.9% vs. 18.0%; <59 vs. 60–69 vs. ≥70; *p* < 0.001) and the proportion of transarterial therapy significantly increased (33.6% vs. 41.0% vs. 48.0%; <59 vs. 60–69 vs. ≥70; *p* < 0.001). In terms of the CPC, the proportion of surgical resection significantly decreased (36.3% vs. 7.9% vs. 0.9%; *A*, *B*, and *C*; *p* < 0.001), while liver transplantation significantly increased with CPC (0.6% vs. 1.5% vs. 11.5%; *A*, *B*, and *C*; *p* < 0.001). Local ablative therapy showed no significant difference with respect to the age group (19.4% vs. 21.4% vs. 18.5%; <59 vs. 60–69 vs. ≥70; *p*=0.784), and a marginal difference was seen for CPC (20.2% vs. 17.7% vs. 16.8%; *A*, *B*, and *C*; *p*=0.094). Local ablative therapy was preferred to surgical resection regardless of age and CPC in tumors ≤2 cm, and surgical resection was preferred to local ablation therapy in tumors >2 cm in all age groups and CPC *A* and *B* (Figures 1 and 2). In patients with tumors >2 cm, the proportion of no treatment increased significantly to 18.1% in cases ≥70 years, 22.1% in CPC B, and 42.9% in CPC *C* (Figures 1 and 2).

### 3.3. PVT-Positive Cases

Of the total 625 patients, 44.1% of patients selected transarterial therapy as the initial treatment, followed by no treatment (33.7%) and systemic chemotherapy (13.7%). RT was selected by 3.0% PVT-positive patients. While the proportion of transarterial therapy decreased significantly with aggravated CPC (56.0% vs. 34.5% vs. 15.4%; *A*, *B*, and *C*; *p* < 0.001), the impact of age on the choice of transarterial therapy was minimal (*p*=0.262) (Table 3). The rate of application of systemic chemotherapy did not change significantly in both age and CPC groups over 10% (*p*=0.292 and *p*=0.235, respectively).

**Figure 3:**
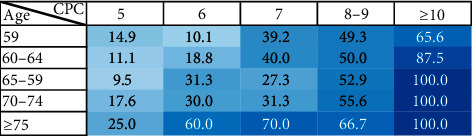
Proportion of patients who did not receive any treatment in the PVT-positive group and with increasing age and a worse Child–Pugh score, the proportion of patients in whom no treatment was increased. The degree of the ratio was expressed in gradation.

In particular, the proportion of patients with no treatment significantly increased with increasing age (29.3% vs. 34.7% vs. 46.4%; <59, 60–69 and ≥70, *p*=0.001) and worse CPC (17.3% vs. 46.8% vs. 75.0%; *A*, *B* and *C*, *p* < 0.001) (Table 3 and Figure 3).

## 4. Discussion

This study examined the treatment preference for HCC with regarding aging and liver function. The Korean cohort showed that cancer treatments were affected by these conditions, which could be related to the fragility of HCC management. Transarterial therapy was preferred to surgical resection in patients with single lesions, and no treatment in our study was preferred for PVT in the elderly and those with worse liver function.

Surgical resection and RFA are recommended by various guidelines for single-lesion HCC [1–6], and TACE is considered optional according to the tumor location and medical comorbidity [4, 6]. TACE could also be used in specific situations such as the use of transplantation as bridging therapy [3]; furthermore, RT, including stereotactic body radiotherapy, is another option for single lesions [1, 4]. However, except for groups <60 years, CPC *A*, and > 2 cm tumor size, TACE was favored in the actual practice of Korean society; in other words, TACE was more frequently used than the recommendations of published guidelines. In a Western survey from France, 81% and 64% of intervention radiologists applied TACE in BCLC A and in combination with other therapies, respectively [12]. Despite the actual outcomes, it should be first considered that surgical resections obtain greater long-term overall survival (OS) and recurrence-free survival than the combination of TACE plus RFA in a meta-analysis [13]. However, in salvage therapy after initial hepatectomy, TACE plus RFA was comparable to repeated surgical resection for OS and morbidity after propensity score matching (PSM) [14]. In another PSM study, TACE plus RFA had better OS than the monotherapy of RFA or TACE in patients with a tumor size <3 cm [15]. In addition, TACE was preferred to systematic chemotherapy in PVT because of the policies of the Korean National Insurance that do not allow changes to TACE after the use of systematic emerging therapies such as sorafenib and, at the end point of our study, did not cover the full costs of these agents. Finally, the solid multidisciplinary approach with the intervention radiologists and hepatologists in each institution had a role in the patients' initial choice of treatment for PVT. In a meta-analysis, TACE plus sorafenib improved the OS, time to progression, and objective response rate with rare moderate adverse events compared to TACE alone [16]. Another PSM study showed similar outcomes with a median 13 months of 1-year OS in the group of TACE plus sorafenib (7 months in group of TACE alone) [17]. Interestingly, a recent Korean randomized control study showed that TACE plus RT was better than sorafenib alone in terms of objective response rate (33.3% vs. 2.2%) and median time to progression (31.0 weeks vs. 11.7 weeks) for macroscopic vascular invasion tumors [18]. A certain rate of TACE in our cohort would be combined with RT when estimating the previous Korean cohort study of RT in PVT, which showed that more than half of the RT was performed with previous TACE [19].

The liver ages with changes in hepatic weight, volume, and blood flow, including bile flow and Kupffer cell function [20]. Several studies have reported that surgical resection is feasible with comparable survival benefit and safety [21–23]. However, a recent meta-analysis showed that the elderly had a slightly lower 5-year OS (55 months vs. 58 months) than younger patients, with an increased hospital mortality of 3.0% (1.2% in younger patients) [24]. Previous systematic reviews have demonstrated that there is no significant difference in morbidity between RFA, TACE, and sorafenib [20, 25]. However, it is important to consider the selection bias that retrospective studies would induce in the absence of randomized control studies regarding the elderly. Thus, a comprehensive geriatric assessment and close examination of liver function before using definitive therapies could better advise the decision of treatment.

In South Korea, HCC primarily develops in patients with hepatitis B and cirrhotic liver. On the other hand, hepatitis C virus- (HCV-) related liver cirrhosis and HCC is a major risk factor in the Western world and Japan [26]. There are many differences in characteristics between HBV and HCV in a Korean Central Cancer Registry study. However, there have been reports of studies that did not show differences depending on the cause of the virus when corrected according to the stage [8]. Since one meta-analysis has shown that the risk of recurrence and survival vary widely in patients successfully treated with HCV-associated HCC, a careful approach should be taken in the treatment of HCV patients [27].

In general, surgeons from Eastern countries adopt a more aggressive surgical approach toward HCC compared to the West in terms of surgical resection or liver transplantation [1–6, 9]. This is because HCC in Eastern patients with HBV occurs more frequently in younger patients and in those who have less severe cirrhosis as opposed to Western patients with HCV, who frequently present with decompensated liver disease. Compared to the West, transarterial therapy is adopted more often than sorafenib for BCLC C patients in Asian countries [9]. Across all stages, first, HCC treatment was most frequently transarterial therapy in North America, Europe, China, and South Korea, percutaneous ethanol injection or radiofrequency ablation in Japan, and resection in Taiwan [26].

In CPC A patients, direct therapy of surgical resection, RFA, TACE, and RT is affordable. The management of patients with CPC B remains a clinical challenge as any intervention might be offset by liver function deterioration [28]. In the survival assessment analyzed using the Korean Central Cancer Registry, the 5-year survival rates of CPC stage A, B, and C patients were 52.1%, 16.6%, and 11.1%, respectively [29]. When considering the use of sorafenib in advanced HCC with CPC B, it is important to conduct careful evaluation of individual patients because the incidence of serious adverse events (AEs) was higher in CPC B patients with a score of 8–9 compared with 7 in GIDEON study [30]. However, the overall incidence of drug-related AEs leading to discontinuation was similar between CPC *A* and *B* patients (17% vs. 21%). In subgroup analysis of Chinese GIDEON, sorafenib was tried in CPC B, which showed that the safety was similar to that in CPC A; however, the OS was worse [31]. In a meta-analysis, the main observations for safety and OS were not significantly different to those in a previous report [32]. In addition, there are reports that sorafenib-related AEs have prognostic significance in OS and/or time to progression (TTP), so this needs to be interpreted carefully [33]. For TACE, the complication was increased in CPC B with a hazard ratio of 2.1 (95% CI, 1.1–4.1) [34]. Furthermore, the albumin-bilirubin score was demonstrated to be a strong predictor of liver decompensation after surgical resection, TACE, and sorafenib [35]. For RT, approximately 20% of radiation-induced liver disease was developed with a median biological effective dose of 56 Gy in CPC *B* [36]. In most guidelines, the best supportive care was strongly recommended for CP score 9 or CPC *C*. Our study showed that 36.3% of patients with single tumors and 75.0% of patients with PVT received no further treatment in CPC *C*.

The cohort of KCCR obtained nationwide population-based data; however, it has limitations regarding conducting customized studies for specific subjects. First, in the current study, the details of subsequent managements combined with initial management were not presented; therefore, TACE will carry greater weighting in the final analysis. Second, we lacked specific information related to fragile conditions such as comorbidity, and only chronological age was assessed in our study. Thirdly, although our study excluded patients with PVT and extrahepatic metastases to evaluate single lesions, some conditions for conducting surgical resection and RFA could have been missed. Lastly, the category of “no treatment” included a minority of patients who visited other hospitals to gather a second opinion, after which they may have chosen further treatment.

## 5. Conclusions

Therapeutic decision-making for HCC is markedly affected by age and liver function in the Korean society. Under the difficult circumference to conduct randomized controlled studies for these fragile patients, previous studies and our own experiences suggest that our observations will assist decision making in actual practice. Experts' consensus would be necessary to further guide the management of elderly patients and those with unfavorable liver function.

## Figures and Tables

**Table 1:** Patient characteristics (*n* = 7559).

Variables	All patients	Single tumor	PVT-positive tumor
	*n* = 7559	*n* = 4633	*n* = 625
*Age*			
Median (range)	60 (21–98)	61 (22–91)	57 (28–92)
≤59	3577 (47.3%)	2182 (47.1%)	366 (58.6%)
60–69	2180 (28.8%)	1355 (29.2%)	145 (23.2%)
≥70	1802 (23.8%)	1096 (23.7%)	114 (18.2%)

*Sex*			
Male	5838 (77.2%)	3471 (74.9%)	527 (84.3%)
Female	1721 (22.8%)	1162 (25.1%)	98 (15.7%)

*Child–Pugh class*			
A	5855 (77.5%)	3850 (83.1%)	327 (52.3%)
B	1422 (18.8%)	667 (14.4%)	241 (38.6%)
C	282 (3.7%)	116 (2.5%)	57 (9.1%)
Hepatitis B	4507 (59.6%)	1736 (37.5%)	413 (66.1%)
Hepatitis C	983 (13.0%)	581 (12.5%)	69 (11.0%)
Non-B, non-C	267 (3.5%)	165 (3.6%)	17 (2.7%)

*ECOG performance status*			
0	4310 (57.0%)	2827 (61.0%)	252 (40.3%)
1	798 (10.6%)	395 (8.5%)	127 (20.3%)
2	154 (2.0%)	51 (1.1%)	38 (6.1%)
3	55 (0.7%)	12 (0.3%)	23 (3.7%)
4	37 (0.5%)	8 (0.2%)	10 (1.6%)
NA	2205 (29.2%)	1340 (28.9%)	175 (28.0%)

*Tumor size (cm)*			
≤2 cm	2746 (36.3%)	2018 (43.6%)	53 (8.5%)
>2 cm	4813 (63.7%)	2615 (56.4%)	572 (91.5%)

*Number of tumors*			
≤4	6511 (86.1%)	4633 (100.0%)	204 (32.6%)
>4	1048 (13.9%)	0 (0.0%)	421 (67.4%)

ECOG, Eastern Cooperative Oncology Group; PVT, portal vein tumor thrombosis; NA, not available.

**Table 2:** Treatment pattern change by age and the Child–Pugh classification in single lesions.

Treatment *n* = 4601	Age (years)			
	≤59	60–69	≥70	*p* value
Surgical resection	859 (39.6%)	390 (28.9%)	194 (18.0%)	<0.001
Liver transplantation	35 (1.6%)	12 (0.9%)	0 (0.0%)	<0.001
Local ablative therapy	422 (19.4%)	289 (21.4%)	200 (18.5%)	0.784
Transarterial therapy	729 (33.6%)	553 (41.0%)	518 (48.0%)	<0.001
Systemic chemotherapy	7 (0.3%)	6 (0.4%)	7 (0.6%)	0.188
Radiation therapy	7 (0.3%)	12 (0.9%)	4 (0.4%)	0.525
No treatment	112 (5.2%)	88 (6.5%)	157 (14.5%)	<0.001

*n* = 4601	Child–Pugh classification			
	*A*	*B*	*C*	*p* value
Surgical resection	1390 (36.3%)	52 (7.9%)	1 (0.9%)	<0.001
Liver transplantation	24 (0.6%)	10 (1.5%)	13 (11.5%)	<0.001
Local ablative therapy	776 (20.2%)	116 (17.7%)	19 (16.8%)	0.094
Transarterial therapy	1405 (36.7%)	356 (54.4%)	39 (34.5%)	<0.001
Systemic chemotherapy	14 (0.4%)	6 (0.9%)	0 (0.0%)	0.282
Radiation therapy	13 (0.3%)	10 (1.5%)	0 (0.0%)	0.010
No treatment	211 (5.5%)	105 (16.0%)	41 (36.3%)	<0 .001

**Table 3:** Treatment pattern change by age and the Child–Pugh classification in PVT-positive patients.

Treatment *n* = 605	Age (years)			
	≤59	60–69	≥70	*p* value
Surgical resection	16 (4.6%)	5 (3.5%)	1 (0.9%)	0.081
Liver transplantation	3 (0.9%)	3 (2.1%)	0 (0.0%)	0.748
Local ablative therapy	3 (0.9%)	1 (0.7%)	1 (0.9%)	0.996
Transarterial therapy	160 (45.6%)	64 (44.4%)	43 (39.1%)	0.262
Systemic chemotherapy	54 (15.4%)	15 (10.4%)	14 (12.7%)	0.292
Radiation therapy	12 (3.4%)	6 (4.2%)	0 (0.0%)	0.137
No treatment	103 (29.3%)	50 (34.7%)	51 (46.4%)	0.001

*n* = 605	Child–Pugh classification			
	*A*	*B*	*C*	*p* value
Surgical resection	20 (6.3%)	2 (0.9%)	0 (0.0%)	<0.001
Liver transplantation	2 (0.6%)	2 (0.9%)	2 (3.8%)	0.094
Local ablative therapy	4 (1.3%)	1 (0.4%)	0 (0.0%)	0.211
Transarterial therapy	178 (56.0%)	81 (34.5%)	8 (15.4%)	<0.001
Systemic chemotherapy	46 (14.5%)	34 (14.5%)	3 (5.8%)	0.235
Radiation therapy	13 (4.1%)	5 (2.1%)	0 (0.0%)	0.060
No treatment	55 (17.3%)	110 (46.8%)	39 (75.0%)	<0.001

PVT, portal vein tumor thrombosis.

## Data Availability

The data used to support the findings of this study are included within the article and are available from the corresponding author upon request.
